# FULLSENSE—An Online Intervention Program for Adults Reporting Sexual Difficulties: A Two-Armed Randomized Controlled Pilot Trial Protocol

**DOI:** 10.1080/19317611.2026.2618264

**Published:** 2026-01-23

**Authors:** Ana Rita Barros, Priscila A. Vasconcelos, Ana Luísa Quinta Gomes, Mariana Carrito, Ana Luísa Patrão, Leonor de Oliveira, Sandra Aguiar, Joana Carvalho, Pedro J. Nobre

**Affiliations:** a Center for Psychology, University of Porto, Porto, Portugal; b Eli Coleman Institute of Sex and Gender Health, University of Minnesota Medical School, Minneapolis, Minnesota, USA; c William James Center for Research, Departamento de Educação e Psicologia, Universidade de Aveiro, Aveiro, Portugal

**Keywords:** Sexual health, sexual dysfunctions, online intervention, randomized controlled trial, feasibility study

## Abstract

**Background:**

Sexuality is a key aspect of human life, significantly influencing health, well-being, and quality of life. Sexual dysfunction may occur throughout the sexual response cycle and is commonly associated with psychological distress and dissatisfaction. Online interventions have proven effective in promoting sexual health by providing accessible, private, and evidence-based support. Cognitive-behavioral therapy (CBT) and mindfulness-based methods show promise in treating sexual dysfunction, enhancing function, increasing satisfaction, and reducing distress. Embracing a comprehensive and positive, pleasure-inclusive view of sexuality is vital for supporting sexual health and overall well-being. This study presents the protocol for a feasibility pilot randomized controlled trial evaluating FULLSENSE, an 8-week, guided online intervention designed to improve sexual well-being in adults reporting sexual dysfunction.

**Methods:**

A two-arm, parallel, open-label design, with the waiting-list control group receiving treatment as usual will be conducted. Participants (N = 70) meeting eligibility criteria will be randomly assigned to the FULLSENSE intervention or a waiting-list control group. Primary outcomes will assess the intervention’s acceptability, usability, and feasibility. Secondary outcomes will evaluate preliminary efficacy in improving sexual satisfaction, functioning, pleasure, and reducing sexual distress, while cognitive and neurophysiological assessments will allow us to explore functional connectivity and correlates of implicit cognition and interoception. Data collection will include self-reported measures and neurophysiology assessments for a nested sample.

**Results:**

Findings will provide insights into the feasibility and preliminary efficacy of FULLSENSE, informing future large-scale trials. If successful, this intervention may facilitate access to sexual health care and support individuals experiencing sexual distress.

**Conclusion:**

The FULLSENSE feasibility pilot trial promises valuable insights into an accessible, evidence-based online intervention for sexual dysfunction, potentially contributing to digital sexual health solutions. The findings of the forthcoming studies will support the intervention’s implementation and ultimately facilitate sexual healthcare access and support.

## Introduction

Sexuality is an essential and intrinsic aspect of human nature. According to the World Health Organization (WHO), sexual health is integral to overall health, well-being, and quality of life (WHO, 2006). Sexual health requires a positive approach to human sexuality and an understanding of the complex factors influencing sexual behavior, with positive impacts on mental, physical, and global well-being (Anderson, 2013; Kolodziejczak et al., 2019; Sladden et al., 2021; Smith et al., 2019; Vasconcelos et al., 2024), and is considered an essential indicator of health across the life course (Ford et al., 2021).

Sexual dysfunction occurs in both men and women and can manifest at various stages of the normal sexual response cycle. Human sexual (dys)function involves both physiological and psychological factors, making it a complex process (Anderson 2013). Sexual dysfunction is often associated with increased levels of psychological and sexual distress, and sexual dissatisfaction (Van Lankveld et al., 2021). There is also growing evidence that interoceptive awareness—the ability to perceive internal bodily states—plays a critical role in sexual functioning. Handy and Meston (2018) (Handy & Meston, 2018) highlight that many women with sexual arousal concerns may not be fully aware of their physiological genital responses. Furthermore, interoceptive awareness is linked to more positive orgasmic experiences in women, including greater frequency and satisfaction (Dixon et al., 2024). Hence, there is a need for treatments focused on enhancing awareness of the bodily sensations associated with sexual arousal.

Among the several therapeutic approaches available, cognitive-behavioral therapy (CBT) and mindfulness-based approaches have been shown to be effective in a range of psychological and sexual dysfunction (Bossio et al., 2019). In recent years, sexual health interventions have increasingly been delivered online. Multiple studies have supported the effectiveness of online interventions for sexual health and have addressed various sexual problems and dysfunctions (Hensel et al., 2022; Van Lankveld, 2016; Weitkamp, Hänisch, & Heesch, 2021). The European Society of Sexual Medicine recently highlighted the fundamental opportunities that online sexual health interventions offer for improving population sexual health (Kirana et al., 2020), given their convenience, privacy, and anonymity (Xue et al., 2024).

Research indicates that online interventions incorporating sexual pleasure can improve sexual health knowledge, attitudes, and behaviors (Borgmann et al., 2023; Bossio et al., 2019). Empirical evidence demonstrates the positive impact of sexual health, including increased happiness and reduced depression, stress, and anxiety 8 (Brody, 2010; Liu et al., 2016; Sladden et al., 2021). It also contributes to social health in relationships, fostering greater satisfaction, intimacy, and commitment (Bossio et al., 2019; Brody, 2010; Diamond & Huebner, 2012).

An online mindfulness-based intervention led to significant improvements in participants’ sexual function, sexual satisfaction and a substantial decrease in distress (Brotto, Stephenson, & Zippan, 2022). Recent research comparing self-led mindfulness and psychoeducation online interventions for women’s sexual desire has demonstrated improvements in sexual functioning, orgasm, pleasure, and sexual distress, with no significant differences between the two approaches (Xue et al., 2024). Extensive literature, primarily focused on women, (Brotto, Stephenson, & Zippan, 2022; Brotto et al., 2021; Mahar et al., 2022; Schover et al., 2020; Stephenson, Zippan, & Brotto, 2021; Stephenson & Meston, 2010; Thomas et al., 2025; Zippan, Stephenson, & Brotto, 2020) have shown the benefits of self-led online sex therapy, including significant pre-post improvements in sexual outcomes, such as increased knowledge about sexuality, greater awareness of thought patterns, and improved perspectives on sexual dysfunction. High satisfaction with these programs suggests their feasibility and potential to help women with sexual dysfunction.

Research with men has shown improvements in intercourse satisfaction and sexual desire. These improvements are facilitated by interventions that address both psychological and physical aspects of sexual health, covering topics such as the importance of sexuality, sexual dysfunction, contributing factors, maladaptive thoughts, genital anatomy, relationship enhancement, mindfulness, and relapse prevention (Banbury et al., 2024; Brotto et al., 2017). Studies have recently demonstrated the efficacy of CBT interventions in improving mental and sexual health (Benzo et al., 2022; Bossio, Higano, & Brotto, 2021; Pieramico et al., 2023). Banbury et al.’s (2021) meta-analysis showed a small to moderate positive effect of Mindfulness-Based Interventions (MBIs) on male sexual dysfunction. Improvements were reported in self-reported erectile function, mindfulness, sexual self-efficacy, and overall well-being during the intervention and at follow-up (Banbury et al., 2024; Brotto et al., 2025).

Despite growing research on online interventions and their potential benefits for sexual health, further investigation is needed to identify the specific mediators and mechanisms responsible for observed improvements (Gorman et al., 2022). Current literature exhibits a scarcity of interventions designed for men without co-occurring health conditions (e.g., cancer), a limited representation of LGBTQ+ populations and the use of mixed-methods approaches integrating mutually informed qualitative and quantitative measures is still limited in research (Kostiukova, Tselenti, & Carvalho, 2026).

Addressing the WHO’s (2006) call for a comprehensive and positive approach to sexuality, encompassing pleasure, sexual thoughts and beliefs, mindfulness, body sensations, body image, sensual exploration, and communication (WHO, 2006), FULLSENSE - an online digitally delivered sexual health intervention program was developed. This intervention program aims to increase knowledge about sexuality, contribute to the resolution of sexual dysfunction, and promote new ways of experiencing and enjoying sexuality. This report presents a research protocol that will support the assessment of FULLSENSE’s acceptability, feasibility, and preliminary efficacy in people reporting distressful sexual complaints compared to a waiting-list control (WLC) condition. The findings of the forthcoming studies will support the intervention’s implementation and ultimately facilitate sexual healthcare access and support. The submission of this protocol is fundamentally justified by its dual methodological value: it establishes scientific transparency through the pre-specification of all hypotheses and analyses to prevent questionable research practices, and it provides a critical blueprint detailing the innovative, integrated (CBT, Mindfulness, Interoception), and scalable methodology of this online intervention for future digital sexual health research.

## Objectives and hypotheses

This study aims to assess the acceptability, usability, feasibility, and preliminary efficacy of FULLSENSE in individuals from the general population with self-reported sexual dysfunction. We hypothesize that FULLSENSE will be well-accepted by participants, demonstrate feasibility for scale-up, and show preliminary efficacy in improving relationship and sexual satisfaction, sexual functioning, reducing sexual distress, and enhancing sexual pleasure.

## Methods

### Study design

A two-arm, parallel, open-label design, with the waiting-list control group, launched in May 2025, with enrollment expected to be concluded by September 2025. This pilot trial will be conducted to identify shortcomings in the study design, evaluate the data collection procedure, and examine the feasibility of the parent randomized controlled trials. This design will also assess the acceptability of FULLSENSE among study participants, the structure and format of the study intervention (i.e., guided), and intraindividual and between-group differences. This trial has received approval from the Ethics Committee of the Faculty of Psychology and Education Sciences, University of Porto (approval number: 2023-09-03f).

### Participants and eligibility criteria

To be eligible for randomization, participants must be adults (≥ 18 years old) of all genders and sexual orientations who have daily access to an internet-connected device, present a distressful sexual complaint over the last 6 months, do not present severe psychopathological (i.e., depression, anxiety), and neurological symptoms, thus not concurrently engaging in therapy (i.e., psychoactive medication) to alleviate sexuality-related issues, or cognitive-behavioral therapy for other psychological issues or in any other clinical study or trial. Participants who are underaged (< 18 years old), or with no internet access, without distressful sexual complaints, or with sexual complaints lasting less than 3 months, presenting severe psychopathological or neurological symptoms, with unstable medication usage over the last 3 months, engaging in sexuality-related other psychological intervention or clinical study or trial, will be excluded. A subsample of 20 participants from the intervention group will be invited to undergo an on-site laboratory data collection, based on the following inclusion criteria: age between (18-40 years), right-handed; reporting enjoyment of pornographic clips with actors of white ethnicity; not using medications or drugs that can interfere with brain activity, not do regular alcohol or drug use, including cannabis. The inclusion criteria for the laboratory sub-sample are based purely on technical and methodological standardization requirements for neurophysiological studies The specific visual stimuli used (featuring actors, typically white) are employed to ensure comparability with standardized, validated stimulus sets. These constraints are fully disclosed and approved by the Ethics Committee.

The total sample size of (35 per arm) is **not intended for statistical power to detect treatment efficacy**. Consistent with recommendations for pilot trials, this sample size is adequate to assess the primary outcomes of **feasibility, acceptability, and usability**, and to provide a robust **estimation of effect sizes and attrition rates** necessary for calculating the required sample size of a future, fully powered definitive trial The study employs a within-subject design with a high number of trials per participant and fine-grained EEG/ERPs measures, which substantially increases statistical power. This approach allows for the detection of subtle intervention-related effects at the individual level and minimizes inter-individual variability. From a statistical standpoint, the large number of repeated measures per participant provides a rich dataset for reliable estimation of within-person changes, even with a smaller N.

### Recruitment, enrollment, and randomization

**Figure 1.**
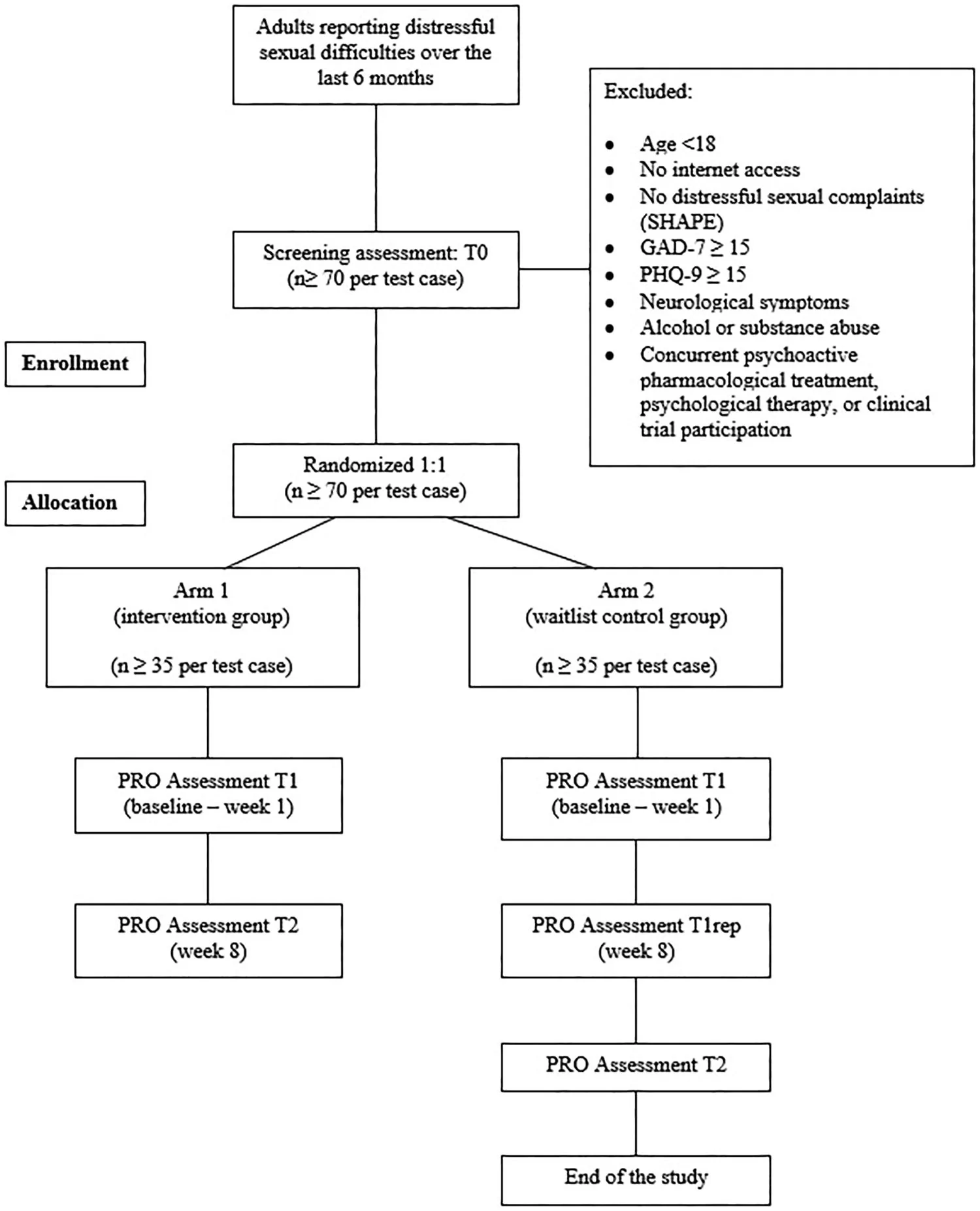
FULLSENSE pilot feasibility randomized controlled trial participant flow diagram. *Note*. PRO = participant-reported outcome.

Potential participants will be identified via patient partner organizations (e.g., the Portuguese Society of Clinical Sexology), university mailing lists, faculty newsletters, patient conferences, and social media platforms. Clinical centers will be notified about the study and supplied with promotional materials that guide interested people to the recruiting website/link for further information and registration. Participants will be invited to participate by filling in an expression of interest survey. A consent form will be executed during registration, followed by the eligibility criteria (T0) screening administered via a telephone interview (LimeSurvey GmbH, n.d.). After completing the online baseline assessment (T1), participants fulfilling all eligibility criteria will receive an informative audiovisual material through email. Such a video will contain a step-by-step guide on how participants will access, use, and complete the program. Maximal procedure randomization will be implemented by a team member using computerized software to create allocation tables (Clinical Trial Randomization Tool, n.d). Participants will be randomly allocated 1:1 to the two study arms (Figure 1). Randomization will also allocate a nested sample (n = 20) to an embedded assessment moment (T1 and T2). This sample will undergo complementary laboratory-based assessments intended to explore potential intervention-related changes in neural activity associated with cognitive and behavioral aspects of sexual response and sexuality in general. Measurements will encompass EEG functional connectivity and exploration of ERPs of sexual implicit cognitions (through an Implicit Association Task). Laboratory tasks will also include a physiological (thermography) plus self-reported body-heat sensations (genital temperature) assessments, intending to explore the relation between both variables as representative of sexual interception abilities.

Based on attrition rates observed in similar 8-week online trials, we anticipate a retention rate of approximately 70–75% (i.e., a predicted attrition of 25–30%) (Zarski et al., 2022). To maximize participant engagement throughout the 8-week period, several retention strategies will be implemented. Specifically, participants will receive regular check-ins and support via email communication with the research team to address any technical or logistical issues and to promote adherence. In addition, weekly automated reminders will be sent to prompt module completion.

### Intervention delivery and assessment

**Figure 2.**
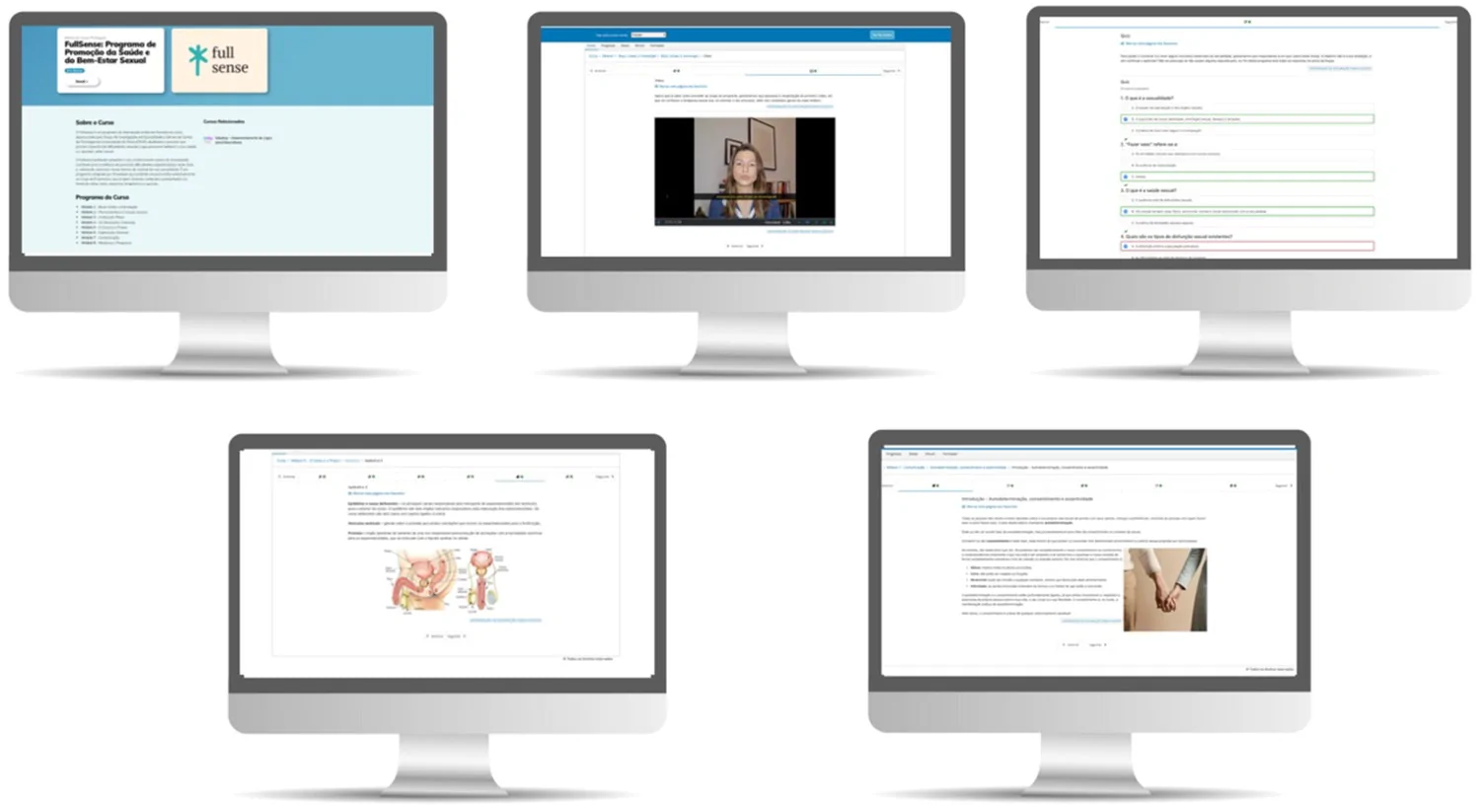
FULLSENSE overview.

FULLSENSE (Figure 2) is an 8-week, digitally delivered online program designed to promote sexual health and well-being in adults experiencing sexual dysfunction through a cognitive-behavioral approach. Participatory design (Bødker & Kyng, 2018) involving secondary end-users (psychologists and sexologists) and agile framework (Hekler et al., 2016), were implemented for program development. The program adopts a transdiagnostic structure featuring sexual health intervention strategies considered appropriate for the different populations under study according to efficacy studies and reviews of literature (Avery-Clark, Weiner, & Adams-Clark, 2019; Berner & Günzler, 2012; Doğan, 2023; Fahami, Pahlavanzadeh, & Asadi, 2015; Günzler & Berner, 2012; Krieger et al., 2023; Mahar et al., 2022; Sharifipour et al., 2024; Zarski et al., 2022). Core components include psychoeducation (Zippan, Stephenson, & Brotto, 2020), cognitive restructuring (Beck, 2020), mindfulness (Brotto, 2013; Brotto, Stephenson, & Zippan, 2022), masturbation training (Both & Laan, 2008; Semans, 1956), communication skills training (Rosenberg, 2002), exercises to heighten sexual sensation awareness and expand erotic repertoire (Brotto, 2013; Brotto, Stephenson, & Zippan, 2022), and sensate focus exercises (Masters & Johnson, 1966). The program is structured into eight weekly modules (Table 1).

**Table 1.** Structure and content of FULLSENSE.

Module	Intervention components	Content
1-Welcome and introduction	Psychoeducation	Welcome and Introduction Basic concepts of sexuality The different sexual dysfunctions Factors that influence sexuality Self-compassion
2-Sexual thoughts and beliefs	Psychoeducation Cognitive restructuring	Cognitive restructuring Sexual scripts and beliefs Thoughts, emotions and behaviors
3-Mindfulness	Psychoeducation Mindfulness	Mindfulness exercises
4-Body sensations	Psychoeducation Mindfulness Sensate focus	Sensate focus/ Interoceptive awareness Spectator syndrome and its impact
5-The body and pleasure	Psychoeducation Sexual skills training Mindfulness	Masturbation training Vulva and Penis’s Anatomy
6-Sensual exploration	Psychoeducation Sexual skills training	Sensual exploration Fantasy and kink
7-Communication	Psychoeducation	Communication Self-determination, consent and assertiveness
8-Change and progress	Relapse prevention	Overview of FULLSENSE and progress evaluation Strategies to maintain progress and prevent setbacks

Regarding human support, FULLSENSE will be delivered using a guided service model, which includes synchronous or asynchronous contacts, support, and feedback from psychologists via email, telephone, and other communication channels. Participants will obtain access to the intervention following preliminary screening assessment and online baseline assessments. The screening assessment (T0) aims to evaluate the fulfillment of the predefined eligibility criteria. In addition to the assessment of age, internet access, and concurrent engagement in psychological therapy or clinical trial, psychopathological symptoms (Patient Health Questionnaire-9 (PHQ-9); Kroenke, Spitzer, & Williams, 2001); Generalized Anxiety Disorder-7 (GAD-7); Spitzer et al., 2006, 57) and sexual dysfunction (Sexual Health Assessment of Practices and Experiences (SHAPE); WHO, 2023) will be evaluated in this initial screening.

After completing the online baseline (T1) assessment, participants meeting eligibility criteria will be registered and randomized, following an appointment with the assigned research member (Psychologist) to provide program access, examine the program’s framework (Figure 2), present the baseline findings, and establish therapeutic goals. The primary outcomes of this pilot trial are acceptability, usability (System Usability Scale (SUS) (Bangor, Kortum, & Miller, 2008) and feasibility measures (i.e., recruitment, randomization, retention, and adherence rates). Acceptability will be assessed in postintervention semistructured interviews. Secondary outcomes will evaluate preliminary efficacy on sexual beliefs (Sexual Dysfunctional Beliefs Questionnaire (SDBQ); Nobre & Pinto‐Gouveia, 2003), sexual satisfaction (Global Measure of Sexual Satisfaction (GMSEX); Lawrance & Byers, 1995), function (Index of Erectile Function (IIEF); Rosen et al., 1997b); and Female Sexual Functioning Index (FSFI); Rosen et al., 1997a), pleasure (Sexual Pleasure Scale (SPS); Sanchez, Crocker, & Boike, 2005) and distress (Female Sexual Distress Scale-Revised (FSDS-R); Derogatis et al., 2002). Additionally, participants will complete a self-report questionnaire to provide their sociodemographic and sexuality-related behavioral data, including age, gender, relationship status, religion, education, sexual orientation, and frequency of sexual activities. Changes in potential transdiagnostic factors, namely interoceptive awareness (Multidimensional Assessment of Interoceptive Awareness (MAIA; Mehling et al., 2012), trauma screening questionnaire (Posttraumatic Stress Disorder Checklist for DSM-5 (PCL-5; Weathers et al., 2013), questions about negative sexual experiences, mindfulness (Cognitive and Affective Mindfulness Scale-Revised (CAMS-R); Feldman et al., 2007), quality of life (World Health Organization Quality of Life: Brief Version (WHOQOL-BREF); The Whoqol, 1998), psychopathology (BSI-18; Derogatis, 2001), and therapeutic outcomes (Outcome Questionnaire (OQ-45); Lambert et al., 2004), will be assessed. As mentioned before, a subsample of participants will be invited to on-site laboratory sessions (at T1 and T2). Using a multimodal approach that combines EEG, behavioral tasks, thermal imaging, and self-report measures on affect (Positive and Negative Affect Schedule (PANAS; Watson, Clark, & Tellegen, 1988) and automatic thoughts (Sexual Modes Questionnaire - Thoughts Subscale (QMS; Nobre & Pinto‐Gouveia, 2003), this complementary part of the study will allow us to explore how brain activity, implicit cognitions, and interoceptive awareness contribute to intervention-related changes in sexual functioning and perception.

Participants will be instructed to conclude each module within a week. Within 24 hours before the pre-defined timeframe for module completion, the assigned research member will evaluate participants’ progress based on the reported results and decide whether participants will have access to the subsequent module. Such results will be shared with the participants, and, if deemed required, further instructions will be provided on the steps to proceed to the forthcoming module. The intervention’s initial effectiveness will be evaluated upon program completion at posttest (T2 for arm 1 and T1rep for arm 2), utilizing the aforementioned measures, and a final interview for debriefing, user-experience and acceptability assessment.

## Data analysis

Descriptive statistics will be provided for demographic characteristics and outcome variables, including means and standard deviations for continuous variables and proportions for categorical variables. Independent sample t-tests and chi-square analyses will be conducted to evaluate potential baseline differences between participants assigned to the experimental and control groups to determine preliminary efficacy. The qualitative data collected in the final interview will be included in the analysis. For the data collected during the lab experiment, we will opt for a multimodal analytical approach. Behavioral and physiological data regarding sexual cognitions and interoception will be collected to explore underlying psychological and neurological processes and potential changes resulting from the intervention. Hence, for the within-subject factors (such as multiple time points or conditions in the ERP or thermal response experiments), rmANOVAs or Linear Mixed Effects (LME) models will be used for detecting overall differences across these conditions. Regression analyses will help in predicting outcomes, and correlations can offer insights into the strength and direction of associations. GMLL will allow us to model the random effects of individuals, accounting for the correlation among repeated observations and providing subject-specific insights. Differences between the experimental and control groups regarding secondary outcomes will also be analyzed. We will comply with the intention-to-treat (ITT) principle for comparisons between treatment groups.

## Results

The results on acceptability, usability, feasibility, and preliminary efficacy of FULLSENSE are expected in the second semester of 2025. Study results will be published in open-access venues according to the Consolidated Standards of Reporting Trials of Electronic and Mobile Health Applications and Online Telehealth guidelines (Eysenbach, 2011). We anticipate that it will be feasible to scale up to parent RCTs and that FULLSENSE will effectively promote positive changes in secondary outcomes.

## Discussion

Sexual dysfunction is often associated with increased levels of psychological distress and sexual dissatisfaction (Handy & Meston, 2018). Online sexual health programs offer a promising avenue for addressing these challenges, providing unparalleled accessibility and scalability. FULLSENSE, a program employing a mixed-methods approach to encompass diverse sexual dysfunction, aims to deepen our understanding of the impact of such interventions.

This study evaluates the feasibility, acceptability, and preliminary efficacy of FULLSENSE, an online intervention designed for adults experiencing sexual dysfunction. This study is designed as a randomized controlled trial (RCT) comparing FULLSENSE to a waiting-list control (WLC) condition. The primary objectives are to assess the feasibility and preliminary efficacy of FULLSENSE in individuals reporting distressful sexual complaints. We hypothesize that FULLSENSE will demonstrate acceptability, usability, feasibility, and preliminary signs of efficacy in improving sexual health, satisfaction, and pleasure. Furthermore, this pilot feasibility trial aims to validate the chosen methodology, ensuring its coherence with participants’ expectations and needs. Individuals experiencing sexual dysfunction often report distress, sexual dissatisfaction, isolation, and other negative impacts on their well-being (Vasconcelos et al., 2024). Effective, accessible, and engaging biopsychosocial interventions are crucial to address these unmet needs.

Digital health interventions (Avery-Clark, Weiner, & Adams-Clark, 2019; Banbury et al., 2024; Benzo et al., 2022; Borgmann et al., 2023; Bossio, Higano, & Brotto, 2021; Bossio et al., 2019; Brotto, Stephenson, & Zippan, 2022; Brotto et al., 2017; 2021; 2025; Van Lankveld, 2016; Zippan, Stephenson, & Brotto, 2020) like FULLSENSE offer a promising avenue for enhancing access to and broadening the dissemination of supportive care. Unlike earlier programs, FULLSENSE does not target a specific population or sexual dysfunction, thus embracing a more comprehensive approach to promoting sexual health. By adopting a positive approach to sexuality that includes pleasure, FULLSENSE addresses a crucial, often neglected aspect of sexual health (Van Lankveld et al., 2021) and aims to be an inclusive program, suitable for all people. An additional distinctive feature pertains to its delivery format, providing a safe and comfortable environment for exploring sensitive content. Regarding the assessment of preliminary efficacy, the inclusion of the nested neurophysiological study using EEG will further investigate whether FULLSENSE has the potential to modulate brain connectivity related to key psychological processes associated with sexual well-being, offering valuable insights into the intervention’s mechanisms of action. Furthermore, a preliminary usability study was conducted to gather feedback from individuals experiencing sexual dysfunction regarding the program’s content, structure, format, and user experience. This process contributed to refining the program, ensuring greater coherence and alignment with the needs of the target population. Also, the program follows a transdiagnostic structure, incorporating multimodal intervention strategies, namely mindfulness-based exercises that address multiple diagnostic categories (Greeson, Garland, & Black, 2014) in different populations experiencing sexual dysfunction. We, also, highlight that the gender and orientation agnostic design is a strength that ensures maximal accessibility to a broad range of adults with sexual difficulties.

As aforementioned, this feasibility pilot trial has several strengths, including its RCT design, the inclusion of diverse sexual dysfunctions (both male and female), the use of validated outcome measures, and its comprehensive and transdiagnostic nature. Nevertheless, some limitations must be acknowledged. While adequate for feasibility testing, the sample size may limit the generalizability of the findings and preclude definitive conclusions about efficacy. Initially designed for evaluative training courses, the platform may not be optimally suited for the program’s learning and reflection exercises, potentially impacting user experience, especially for smartphone users. Consequently, recruitment and retention of participants may present challenges, so future optimization of the platform for smartphone use might also be crucial. A key limitation of this protocol, inherent to its pilot nature, is the **small sample size (N = 70)**. While sufficient for assessing feasibility and generating preliminary effect sizes, the study is **not powered to draw definitive inferential conclusions** regarding the efficacy of the FULLSENSE intervention. The results must therefore be interpreted cautiously and strictly within the context of **viability and preparatory data** for a larger trial Also, the participatory design approach did not include its primary end users, which may influence acceptability and feasibility. We acknowledge the limitation that, due to its general design, the program may not fully reflect the specific, nuanced experiences of all sexual and gender minority groups, which may influence individual engagement. We clarify that future qualitative research will be essential to explore these differences in depth.

The FULLSENSE feasibility pilot trial promises valuable insights into an accessible, evidence-based online intervention for sexual dysfunction, contributing to digital sexual health solutions. The mixed-method approach will deepen understanding of FULLSENSE’s acceptance and implementation. By adopting a comprehensive approach to sexual health, and sexual dysfunction in particular, the FULLSENSE feasibility pilot trial might ultimately contribute to realizing sexual health as a fundamental right (Van Lankveld et al., 2021). Upon completion of the **FULLSENSE Pilot RCT**, the research team is committed to preparing a separate follow-up manuscript reporting the **feasibility outcomes, participant engagement metrics, and preliminary effect sizes**, with the goal of submitting it to a high-impact journal immediately. This sequential dissemination plan ensures that the scientific community first benefits from the **transparent methodology (Protocol)** and subsequently gains access to the **empirical results** necessary for planning a future, fully powered trial.
